# Infrared measurements of glucose in peritoneal fluid with a tuneable quantum cascade laser

**DOI:** 10.1364/BOE.393617

**Published:** 2020-06-18

**Authors:** Ine L. Jernelv, Dag Roar Hjelme, Astrid Aksnes

**Affiliations:** Department of Electronic Systems, Norwegian University of Science and Technology (NTNU), O.S. Bragstads plass 2A, 7491 Trondheim, Norway

## Abstract

Fast and accurate continuous glucose monitoring is needed in future systems for control of blood glucose levels in type 1 diabetes patients. Direct spectroscopic measurement of glucose in the peritoneal cavity is an attractive alternative to conventional electrochemical sensors placed subcutaneously. We demonstrate the feasibility of fast glucose measurements in peritoneal fluid using a fibre-coupled tuneable mid-infrared quantum cascade laser. Mid-infrared spectra (1200–925 cm^−1^) of peritoneal fluid samples from pigs with physiological glucose levels (32–426 mg/dL, or 1.8–23.7 mmol/L) were acquired with a tuneable quantum cascade laser employing both transmission and attenuated total reflection (ATR) spectroscopy. Using partial least-squares regression, glucose concentrations were predicted with mean absolute percentage errors (MAPEs) of 8.7% and 12.2% in the transmission and ATR configurations, respectively. These results show that highly accurate concentration predictions are possible with mid-infrared spectroscopy of peritoneal fluid, and represent a first step towards a miniaturised optical sensor for intraperitoneal continuous glucose monitoring.

## 1. Introduction

Challenges related to chronic illnesses, an ageing population, and increased healthcare costs have led to great interest in development of sensor solutions that enable real-time or point-of-care (POC) monitoring, such as glucose sensors for diabetic patients. Monitoring of blood glucose levels (BGLs) is crucial for treatment of diabetes in order to avoid short- and long-term effects related to low (hypoglycaemia) and high (hyperglycaemia) glucose levels for patients treated with insulin [1–3]. Most commercially available glucose sensors are electrochemical and estimate glucose concentrations based on an enzymatic reaction, either with fingerprick measurements or with a continuous glucose monitoring (CGM) device worn on the body [4].

Optical measurement methods have a large potential for CGM, as they may be fast and reagent-free [5]. Mid-infrared (MIR) spectroscopy has several characteristics that are advantageous for POC monitoring. The MIR range, 2.5-25 μm, covers the fundamental vibrational and rotational absorption bands of organic and inorganic molecules. MIR spectroscopy can thereby produce relatively strong absorption bands with uniquely identifiable features. The commercial availability of tuneable quantum cascade lasers (QCLs) has also opened up more avenues for MIR spectroscopy applications [6]. QCLs are small, with high spectral power density, and can be engineered to cover a wanted wavelength region [7]. Tuneable QCLs have been used for in vitro measurements of e.g. human blood serum [8], and promising preliminary investigation of subcutaneous glucose measurements in rats has been shown with a single-wavelength QCL [9]. For non-invasive approaches, Werth et al [10]. showed glucose measurements based on backreflected light from the hand, while Kino et al [11]. determined glucose concentrations from measurements of the inner lip mucosa.

CGM devices used today are placed with a patch on for example the arm or the abdomen, with a small filament positioned under the skin. Through this, the glucose concentration is measured in the interstitial fluid (ISF) between cells. This approach often improves control over the BGL compared to fingerprick measurements as the user typically gets glucose measurements every 5 minutes [12]. However, measuring glucose in the ISF is not without disadvantages. Glucose molecules have to diffuse from capillaries to the ISF, in addition to the time constant between the tissue and the sensing site. Further uncertainties are introduced with changes in tissue dynamics, such as temperature and pressure in the region, as well as biofouling and encapsulation of the sensor due to an inflammatory response [13]. Subcutaneous (SC) glucose levels have been reported to lag behind the BGL by 5-15 minutes, which can be detrimental to glucose control [14–16]. Similar delays have also been found for various non-invasive approaches. This might be a real limit to further advancement of diabetes treatment. An artificial pancreas (AP), which would be a fully automated system with glucose measurements and insulin infusions, would depend heavily on detecting rapid changes in the BGL [17,18].

The abdominal cavity, or the peritoneal space, has been suggested as a potential site for glucose measurements and insulin infusions [19–21]. Peritoneal fluid, which lubricates the organ surfaces in the peritoneal space, contains glucose in similar concentrations to the BGL. Promising short-term studies with intraperitoneal (IP) glucose sensing using electrochemical sensors have been reported [22], and some preliminary data from recent long-term studies have been demonstrated [23]. These studies have shown faster glucose sensing dynamics in the peritoneal space compared to SC sites, indicating that the intraperitoneal space may be a better site for a fully automated AP. Peritoneal fluid may be considered an ultrafiltered plasma, with lower cellularity and lower total protein content than e.g. serum or ISF. We therefore expect the peritoneal cavity to be an attractive site for mid-infrared spectroscopic CGM. Glucose sensing in peritoneal fluid with mid-infrared spectroscopy has so far not been investigated.

In this study we demonstrate the feasibility of fast and accurate glucose measurements in peritoneal fluid from pigs using a fibre-coupled mid-infrared quantum cascade laser. The compositions of pig blood and human blood are similar [24,25], and the optical properties are expected to be similar. Due to this, and the general use of pigs as research animals, the results presented here should be transferable to humans. Highly accurate predictions of glucose levels in unknown fluid samples were achieved using regresssion methods on the MIR spectra. Comparable results were achieved both in transmission and attenuated total reflection (ATR) configurations using two fibre-coupled setups developed with the aim of future in vivo applications. This demonstrates the potential of mid-infrared spectroscopy as an optical measurement method for glucose monitoring in the peritoneal space.

## 2. Materials and methods

### 2.1 Biological samples

Peritoneal fluid and blood samples were collected from anaesthetised pigs that were used in animal trials from November to December 2019 at the Department of Clinical and Molecular Medicine, NTNU, Trondheim. The animal experiments were approved by the Norwegian Food Safety Authority (FOTS number 12948), and were in accordance with "The Norwegian Regulation on Animal Experimentation" and "Directive 2010/63/EU on the protection of animals used for scientific purposes". Samples were acquired from a total of six pigs. The animals were a mixture of male and female, and weighed 40-60 kg. No additional animals were sacrificed for these fluid measurements.

In total, 79 centrifuged peritoneal fluid samples from five different pigs, and 21 blood samples from two different pigs were obtained. Sample details are summarised in Table 1. In addition, five non-centrifuged peritoneal samples were acquired from one pig for comparison with the centrifuged peritoneal samples. Samples were kept in Eppendorf tubes with 1.5 mL volume and stored at -18°C. Most of the peritoneal samples were centrifuged to remove excess blood residue and coagulate material, but were otherwise untreated. The non-centrifuged peritoneal samples were heparinised in order to prevent coagulation. The blood samples were heparinised and centrifuged, and the resulting blood plasma samples were used for measurements.

**Table 1.** Overview of fluid samples from pigs.

Sample type	Pig #	No. samples	Initial glucose levels	Spiked glucose levels
Peritoneal (centrifuged)	1-3, 5, 6	79	33–140 mg/dL	32–426 mg/dL
Peritoneal (not centrifuged)	4	5	72–90 mg/dL	75–200 mg/dL
Blood plasma	4,5	21	65–105 mg/dL	65–346 mg/dL

The acquired samples were measured in a blood gas analyser (ABL 725, Radiometer ApS, Brønshøj, Denmark) to get accurate reference measurements for the glucose concentrations. The samples had an initial glucose concentration range of 33–140 mg/dL (1.8–7.8 mmol/L) for the peritoneal samples, and 65–105 mg/dL (3.6–5.8 mmol/L) for the blood plasma samples. A subset of samples were spiked in order to investigate the entire range of physiological concentrations found in humans. Spiking was performed by making a highly concentrated glucose solution in demineralised water, and adding an appropriate amount to each sample with a micropipette. This added less than 5% extra volume to each sample, and the calculated spiked concentrations were adjusted according to the additional volume. The final concentration ranges after spiking were 32–426 mg/dL (1.8–23.7 mmol/L) for the peritoneal fluid samples and 65–346 mg/dL (3.6–19.2 mmol/L) for the blood plasma samples. Lactate and electrolyte levels were also measured in the blood gas analyser, while total protein and albumin levels were measured in a selection of the samples at an external laboratory. The levels of these other analytes are summarised in the Appendix.

### 2.2 Data analysis

Multivariate data analysis for concentration prediction was done with the SpecAnalysis software, which can be found on GitHub (https://github.com/jernelv/SpecAnalysis). For the centrifuged peritoneal samples, the data was divided into a training set with 48 samples and a validation set with 31 samples. Samples from pig 1, 3, and 5 were used for training, and samples from pig 2 and 6 were used for the validation set. An optimal regression model was found by performing cross-validation (CV) on the training set. CV was done with random subsets (10 splits, 20 iterations). The best model from the training data was then applied to the validation data. A similar approach was used to analyse the subset of peritoneal samples that were not spiked. This subset consisted of 26 samples, with 14 samples used as a training set and 12 samples used as a validation set. The blood plasma dataset was not divided into training and validation sets, as the total dataset was 21 samples and the samples were obtained from only two pigs. Instead, leave-one-out cross-validation (LOOCV) was used to evaluate the prediction accuracy.

Modelling was done with partial least-squares (PLS) regression, a method for multivariate analysis that combines dimensionality reduction and linear regression. PLS regression with 3–12 latent variables (LVs) was evaluated with cross-validation.

Different pre-processing methods were used on the datasets. Data is always mean-centered prior to PLS regression, so this method was applied to all data. Different filter/smoothing methods (Savitzky-Golay filter, Fourier filter), spectral derivatives (first and second derivatives), background correction, and normalisation were tested on the data. All possible combinations of the included methods were tested in order to find the optimal pre-processing steps, and further details can be found in the Appendix.

The regression models were evaluated with the mean absolute percentage error (MAPE), which is defined as:

(1) $$\text{MAPE}=\frac{1}{n}\sum_{t=1}^{n}|\frac{y_{t}-{\hat{y}}_{t}}{y_{t}}|$$

where $y_{t}$ are the real concentration levels and ${\hat{y}}_{t}$ are the predicted concentration levels, with n observations. MAPE is a common method used to evaluate regression models, and is independent of scale. MAPE is also referred to as mean absolute relative difference (MARD), especially for CGM systems. However, many other measures for prediction accuracy are also used, so for completeness the root-mean-square error (RMSE), the coefficient of determination (R^2^), and the standard error of prediction (SEP) are reported in the Appendix.

### 2.3 Experimental setup

**Fig. 1.**
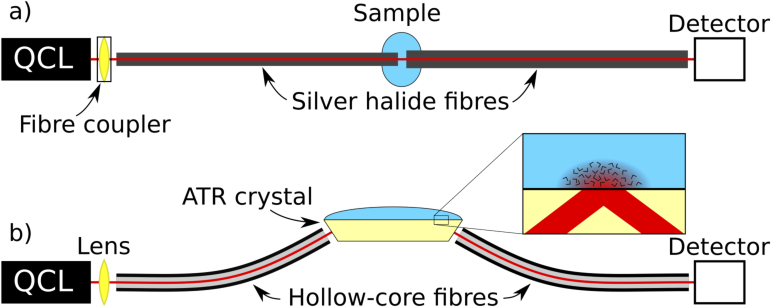
Schematic illustration of the two experimental setups used for this study. a) Transmission spectroscopy setup. b) ATR spectroscopy setup. The inset illustrates light propagation and total internal reflection in an internal reflection element (IRE), which is the principle for evanescent field sensing.

In this study we performed measurements in two experimental setups based on transmission spectroscopy and attenuated total reflection (ATR) spectroscopy, see Fig. 1. An external-cavity quantum cascade laser (EC-QCL, Hedgehog-UT, Daylight Solutions Inc., USA) was used as a light source in both setups. These setups were similar to those we previously characterised and used for measurements of aqueous solutions, see Ref. [26] for further details. The full tuning range of this laser was 1200–900 cm^−1^, which corresponds to 8.3–11.1 μm. The EC-QCL ran in pulsed mode at a frequency of 100 kHz with 500 ns long pulses. An MCT detector (PCI-4TE, VIGO System S.A.) with thermoelectric cooling was used to collect the infrared radiation. Spectra were acquired in the 1200–925 cm^−1^ range, and the total measurement time for one raw spectrum was 10 s. The acquired measurements were calculated as absorbance ($A=-\log I/I_{0}$), based on the intensity measured with a sample (I) and the intensity measured with a reference ($I_{0}$). Deionised water was used for the reference spectra.

Silver halide fibres (ART photonics GmbH, Germany) were used to guide light in the transmission setup, and a gap between the fibres was used for sensing. The gap between the fibres was 200 μm, in accordance with the optimal pathlength found previously for our setup [27]. 150 μL fluid was used for each measurement.

A trapezoidal ZnS prism was used as an internal reflection element (IRE) in the ATR setup. This prism had a surface area of 24 mm x 2.4 mm, and a height of 1.2 mm. The thickness of this prism was half of that used in our previous ATR experiments [26], and it had 11 reflections on the top surface. Hollow-core optical fibres with an inner diameter of 1 mm were used for in- and out-coupling of light. These fibres had an inner silver coating, which was made in a chemical silver-mirror reaction [28,29]. The larger hollow-core fibre could efficiently collect radiation from the end facet of the IRE without the need for additional focussing optics. 100 μL fluid was necessary for each measurement.

ATR spectroscopy is based on absorption of the evanescent field that arises in total internal reflection (TIR) between media with high and low refractive indices, see the inset in Fig. 1(b) for an illustration of the concept. The evanescent field is non-propagating, but can still interact resonantly with absorbing analytes. The absorbance signal correlates with the proportion of the field emanating from the crystal, and the absorbance increases approximately linearly with the number of reflections [30].

## 3. Results

### 3.1 Transmission setup

**Fig. 2.**
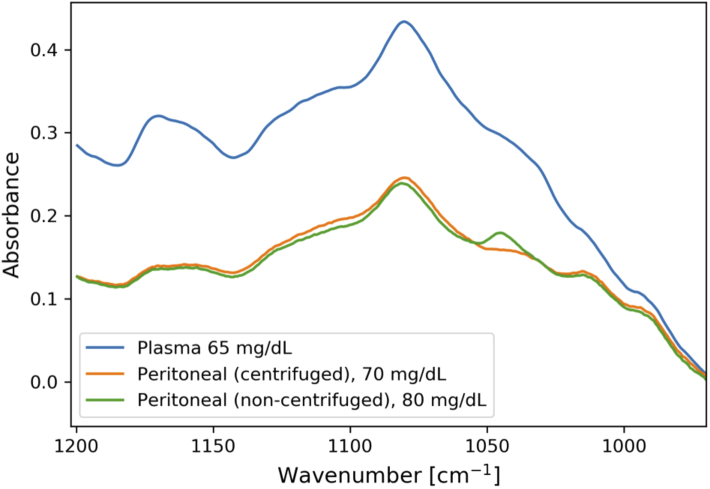
Transmission spectra of peritoneal fluid (centrifuged and non-centrifuged) and plasma samples with similar glucose concentrations for comparison. The samples are all from different individuals.

Spectra acquired from the three sample types that were measured (centrifuged and non-centrifuged peritoneal fluid, and blood plasma) are shown in Fig. 2 for comparison. These spectra were background-corrected, but otherwise untreated. The displayed spectra have similar glucose concentrations and are from unspiked samples from different individuals. The spectra are plotted for the range 1200–970 cm^−1^ for clarity, as the lower wavenumbers did not contain any visible spectral features. The non-centrifuged peritoneal fluid samples were acquired mainly for this comparison. These samples were not used further for regression analysis, as there were only five non-centrifuged samples in total.

The two types of peritoneal fluid samples had very similar spectra, with a difference in the absorption feature at approximately 1045 cm^−1^. The non-centrifuged peritoneal fluid samples contained blood residue, and were found to have coagulate material that formed over time despite treatment with heparin as an anticoagulant. Spectra from the plasma samples had the same general shape as the peritoneal fluid spectra, but with a higher total absorbance. The non-centrifuged peritoneal fluid and blood plasma samples were heparinised, which could potentially also have affected the spectra. However, heparin does not have any notable absorption features in the wavelength range of the laser at the concentrations used [8], so this was not expected to affect the measurements.

**Fig. 3.**
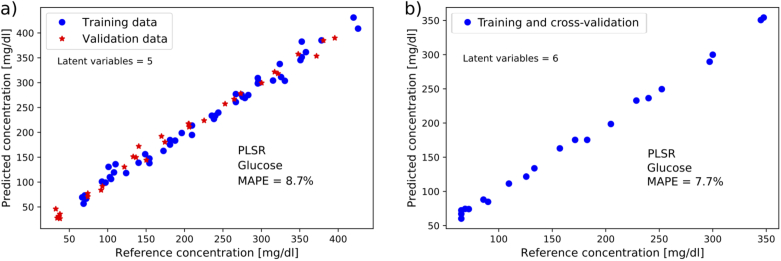
Results from multivariate analysis of the transmission spectroscopy data. a) Prediction of glucose concentrations in spiked and unspiked samples of peritoneal fluid from pigs (48 samples for training, 31 samples for validation). b) Result from cross-validation of glucose prediction in spiked and unspiked samples of pig blood plasma (21 samples).

The predicted glucose concentrations in peritoneal fluid and blood plasma measured in the transmission setup are shown in Fig. 3, analysed with PLS regression using 5 and 6 LVs, respectively. For the peritoneal samples, the training data had an internal MAPE of 8.3% with cross-validation. For the 31 samples that were used as validation data, the MAPE was 8.7%. As a comparison, the MAPE for only the unspiked samples with concentration range 33-140 mg/dL was 6.1%. The blood plasma dataset was smaller at 21 samples in total, and therefore only cross-validation was done on this data. The MAPE for plasma was 7.7%. These results were achieved with a Savitzky-Golay filter for pre-processing (width 9, order 1). No additional pre-processing was used for this data.

### 3.2 ATR setup

**Fig. 4.**
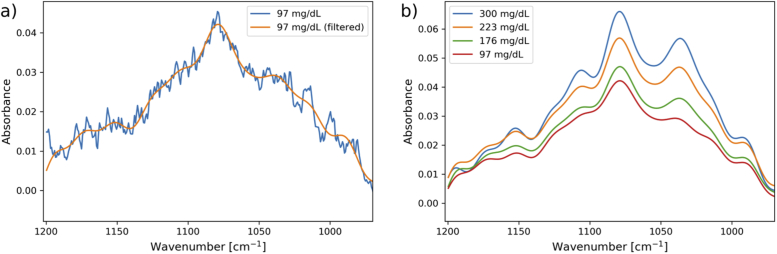
a) Spectrum from ATR spectroscopy of a peritoneal fluid sample before and after Fourier filtering, and b) Filtered ATR spectra of peritoneal fluid samples with different glucose concentration levels.

The acquired ATR spectra were affected by a high frequency noisy structure, most likely due to reflections/interferences in the setup. The intensity of these features was minimised through alignment, but was difficult to eliminate completely. However, as the effect on the spectra was constant between measurements, the concentration prediction was only minimally affected. The high frequency features could also be removed with filtering methods, and the example in Fig. 4(a) shows the result of applying a Fourier filter with a Blackman-Harris window function (cutoff at 28 datapoints in Fourier space, window size multiplier at 1.3).

Example spectra of peritoneal samples with glucose concentrations ranging from 97-300 mg/dL are plotted in Fig. 4(b). The spectra have noticeable changes in absorption features with increasing glucose concentrations, in particular around the 1035 cm^−1^ glucose absorption peak.

**Fig. 5.**
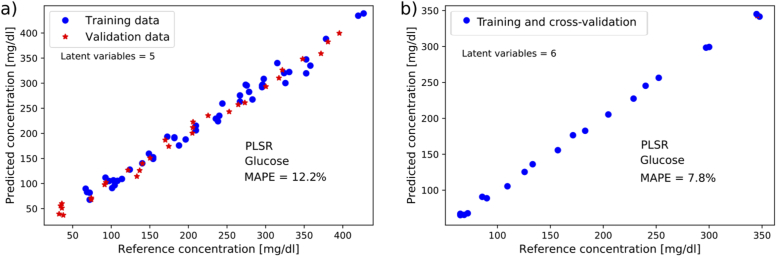
Results from multivariate analysis of the ATR spectroscopy data. a) Prediction of glucose concentrations in spiked and unspiked samples of peritoneal fluid from pigs (48 samples for training, 31 samples for validation). b) Result from cross-validation of glucose prediction in spiked and unspiked samples of pig blood plasma (21 samples).

For the ATR measurements, the predicted glucose concentrations for peritoneal fluid and plasma samples are shown in Fig. 5. The achieved MAPE for glucose in peritoneal fluid was 12.2%, and for blood plasma samples the MAPE for cross-validation was 7.8%. As in the transmission measurements, the optimal models were found using 5 LVs for the peritoneal fluid measurements and 6 LVs for the blood plasma measurements. Application of a Fourier filter with the same parameters as in Fig. 4 gave the lowest prediction errors. For the smaller dataset of unspiked peritoneal fluid samples, the achieved MAPE was 10.5%.

## 4. Discussion

Highly accurate prediction of glucose concentrations in peritoneal fluid was achieved with QCL-based spectroscopy with 8.7% and 12.2% MAPE in the transmission and ATR configurations, respectively. This is an encouraging preliminary result, as the accuracies of commercial electrochemical CGM systems are in the range 9–20% [31,32]. The results are very promising for fast and accurate glucose prediction as the measurement time was only 10 seconds, compared to electrochemical sensors that usually average over 5 minutes. Rapid measurements can potentially contribute to improvements in the health outcomes of diabetes patients, as long-term complications can be reduced if the BGL is measured continuously with high accuracy and short response time. Concentration prediction was also accurate for low glucose concentrations (<100 mg/dL), which is a critical concentration range for management of hypoglycaemia in diabetic patients. This is especially notable because the lowest concentration in the training set was 72 mg/dL, while the lowest concentration was 32 mg/dL in the validation set. Hence, the features in the measured spectra were still interpretable at very low concentrations, and the regression model could be extended to unknown concentrations. All measurements used for the validation set were from different pigs than the data in the training set, which also showed that accurate predictions could be made with samples from different individuals. Accurate predictions through cross-validation were also found for the blood plasma samples. The separate analysis of the unspiked peritoneal samples also demonstrated that glucose concentrations could be accurately determined in natural peritoneal fluid. The lower prediction errors compared to the full analysis, at 6.1% and 10.5% for transmission and ATR spectroscopy, respectively, can be attributed to the smaller dataset and narrower concentration range.

The prediction error was somewhat worse in the ATR setup. This was likely due to a decrease in SNR due to a lower absorbance signal, and the presence of a high-frequeny noisy structure over the absorbance spectrum. The exact origin of this signal interference was not determined, but was probably related to reflections/interferences in the setup. Spectra with the same shape as in the transmission measurements could be recovered through Fourier filtering. With in vivo applications in mind, the multireflection prism used as an IRE will likely be too large and bulky for a practical implantable sensor. One option is to use a smaller single-reflection IRE with signal-enhancement, which has given good preliminary results in the same setup as used here [33]. As discussed in our earlier study [26], a commercialised QCL-based sensor will also likely require the use of a few fixed-wavelength lasers for cost and size reduction instead of a tuneable laser. Some previous QCL studies on glucose measurements have explored concentration predictions with only a few wavenumbers [34,35], which has demonstrated that this is a possible avenue for sensor development. These fibre-coupled setups are otherwise highly suitable for further development towards in vivo measurements.

The peritoneal fluid acquired directly from the pig trials was contaminated with blood residue, which was introduced by the abdominal incisions for placement of sampling tubes and a bladder catheter. Blood is not naturally found in peritoneal fluid, and was deemed as unwanted for further measurements. Most of the peritoneal samples were therefore centrifuged prior to measurements. This removes the blood cells, but also potentially white blood cells and larger proteins that are found naturally in the peritoneum. A few non-centrifuged peritoneal samples were therefore measured in order to investigate the difference between these sample types. The spectra of centrifuged and non-centrifuged samples were very similar, with the main difference being a stronger absorption feature in the non-centrifuged samples at 1045 cm^−1^ (Fig. 2). Absorption around 1035 cm^−1^ is an important feature of glucose, and the additional feature in non-centrifuged peritoneal fluid could potentially influence glucose concentration prediction. However, other fluids with larger interferences around 1035 cm^−1^, such as blood serum, have previously been investigated with accurate glucose prediction [8], and the prediction of glucose concentration should therefore not have been unduly affected by using centrifuged samples. The origin of this absorption feature was also uncertain, as it could be a product of either blood residue or other analytes that were removed by centrifugation. The non-centrifuged samples were more difficult to work with due to formation of coagulate material, despite the addition of an anticoagulant, and the exact spiking concentration was more uncertain. It was therefore determined that centrifuged peritoneal samples were acceptable for this initial study.

Peritoneal fluid spectra had the same general shape as the blood plasma spectra, but the total absorbance signal was higher for the blood plasma spectra. This indicates a higher concentration of absorbing analytes in plasma compared to peritoneal fluid. This is in agreement with previous research, which has shown lower concentrations of e.g. albumin and lactate in bovine peritoneal fluid compared to bovine plasma [36]. This was also shown from the measurements of total protein and albumin in a subset of the samples, which was done at an external facility (see Table 2 in the Appendix). Total protein concentrations were more than twice as high in the blood plasma samples compared to the peritoneal samples, and the albumin concentrations were also found to be higher in blood plasma. Total protein and albumin concentrations in the non-centrifuged peritoneal samples did not differ considerably from the centrifuged samples. This indicates that protein concentrations were not meaningfully affected by centrifugation, although this is not a definitive conclusion as the non-centrifuged samples were from only one animal.

Although a total of 79 centrifuged peritoneal fluid samples were measured, these were obtained from only 5 individual pigs, and the initial glucose levels were in a relatively narrow range. The amount of peritoneal fluid in the body is limited (<50 mL), which restricts how much and how often peritoneal fluid can be sampled. A broader concentration range was achieved by spiking a subset of samples. Spiking is an efficient way to get a more varied sample set, however it does not necessarily reflect real-life variations. Peritoneal fluid is a complex matrix where several components are in equilibrium to some degree, and a change in glucose concentrations will usually occur together with a change in e.g. electrolyte levels. This could not be recreated with concentration spiking. Some between-individual and within-individual variation in the samples might therefore have been missed. However, the validation results were very good considering the large range of glucose concentrations. In addition, the samples used for validation were from different pigs than the ones that were used for training the regression model, which shows that concentration predictions with high accuracy can be achieved across individuals.

## 5. Conclusion

QCL-based spectroscopy setups were used for direct and reagent-free measurements of glucose levels in peritoneal fluid. Both direct transmission spectroscopy and evanescent field sensing were accurate for glucose level prediction, with achieved MAPEs of 8.7% and 12.2%, respectively. Spectra acquired from peritoneal fluid were found to be similar to those from blood plasma, but with less total overall absorbance.

This study demonstrates that glucose concentrations can be measured accurately in peritoneal fluid, with similar results to previous studies using mid-infrared spectroscopy for measurements of human blood serum [8]. Intraperitoneal sensing has been suggested as a faster and more accurate way of controlling glucose levels in diabetic patients. Future work should include in vivo measurements in an animal model to confirm the functionality of this sensor system in realistic sensing scenarios.

## Appendix

### Analyte overview

Table 2 summarises the fluid components that were measured in the peritoneal fluid samples and the blood plasma samples. Glucose, lactate, and electrolyte (Na^+^, K^+^, Ca^2+^, Cl^−^) levels were measured in in an ABL 725 blood gas analyser (ABL 725, Radiometer ApS, Brønshøj, Denmark). These measurements were done directly after each pig trial was finished, and the samples were subsequently frozen at -18^∘^C. In addition, a selection of samples were sent to Sentrallaboratoriet, Faculty of Veterinary Medicine, Norwegian University of Life Sciences (NMBU) for measurements of total protein and albumin. Total protein concentration was measured with the Biuret method in an Advia 1800 Chemistry System (Siemens Healthineers AG, Germany). Albumin concentration was measured by capillary electrophoresis in a Sebia Capillarys 2 (Sebia GL, France). As total protein and albumin were measured in only a subset of the samples, it is likely that the concentrations ranges found were not completely representative of the actual variance in the samples.

**Table 2.** Overview of analyte concentrations in the different fluid types obtained from pig trials.

	Fluid type		
Analyte	Peritoneal (centrifuged)	Peritoneal (non-centrifuged)^*a*^	Blood plasma
Glucose	33–140 mg/dL	72–90 mg/dL	65–105 mg/dL
	(1.8–7.8 mmol/L)	(4–5 mmol/L)	(3.5–4.9 mmol/L)
Lactate	1.1–2.4 mmol/L	1.4–1.8 mmol/L	0.4–1.8 mmol/L
Na^+^	134–138 mmol/L	132–135 mmol/L	131–138 mmol/L
K^+^	3.9–5.2 mmol/L	5.3–5.5 mmol/L	4–4.7 mmol/L
Ca^2+^	1.1–1.3 mmol/L	1.2 mmol/L	1.2–1.35 mmol/L
Cl^−^	98–104 mmol/L	99–102 mmol/L	97–103 mmol/L
Total protein^*b*^	15–21 g/L	16–17 g/L	42–46 g/L
Albumin^*b*^	7–11 g/L	7–8 g/L	16–18 g/L

^*a*^ Measured from only one individual

^*b*^ Concentration ranges for total protein and albumin were obtained from a subset of samples, and are therefore likely not representative of the full variance in all the samples measured.

### Pre-processing methods

The measured mid-infrared spectra consisted of 390 datapoints in the 1200-925 cm^−1^ range and had a resolution of 0.7 cm^−1^. Pre-processing methods were tested on the raw spectra as a part of the data analysis, and the variants tested are detailed in Table 3.

**Table 3.** The following variants of pre-processing methods were tested in this study, and all possible combinations were evaluated.

Method	Variants
Baseline correction	None, constant, linear
Savitzky-Golay filter	Filter width: 3, 5,…, 21 data points
	Polynomial order: 1st, 2nd, or 3rd
Fourier filter	Window function: none, Blackman-Harris, Hamming, Hann
	Filter cutoff: 20, 21,…, 50 data points in Fourier space
	Filter window size: 1.1, 1.2, 1.3
Spectral derivative	No derivative, 1st, or 2nd derivative
Normalisation	Scaling, standard normal variate, multiple scatter correction

### Prediction accuracy

Various measures of prediction accuracy are used in multivariate analysis. Table 4 summarises four different measurement errors from the analysis of peritoneal fluid and blood plasma in the QCL-based transmission and ATR spectroscopy setups. These are mean absolute percentage error (MAPE), root-mean-square error of prediction or cross-validation (RMSEP/RMSECV), coefficient of determination (R^2^), and standard error of prediction (SEP). Training and validation sets were used for the peritoneal fluid samples, and the prediction accuracies are based on the results on the validation data. Analysis of the unspiked peritoneal fluid samples used a subset of the total datasets, with 14 samples in the training set and 12 samples in the validation set. As the plasma dataset was smaller, it was evaluated as a training set with leave-one-out cross-validation (LOOCV).

**Table 4.** Overview of various prediction errors obtained for multivariate analysis of glucose levels in peritoneal fluid and blood plasma from pigs.

Measurement	MAPE	RMSE	R^2^	SEP
Peritoneal, transmission	8.7%	9.9 mg/dL	0.9926	10 mg/dL
Peritoneal, ATR	12.2%	12.8 mg/dL	0.989	11.8 mg/dL
Unspiked peritoneal, transmission	6.1%	5.5 mg/dL	0.9845	5.3 mg/dL
Unspiked peritoneal, ATR	10.5%	9.6 mg/dL	0.9621	9.4 mg/dL
Plasma, transmission	7.7%	9.2 mg/dL	0.9915	9.1 mg/dL
Plasma, ATR	7.8%	9.4 mg/dL	0.9899	9.3 mg/dL
